# Venom Toxins as Potential Targeted Therapies

**DOI:** 10.3390/toxins11060338

**Published:** 2019-06-13

**Authors:** Hang Fai Kwok

**Affiliations:** 1 Cancer Centre, Faculty of Health Sciences, University of Macau, Taipa, Macau, China; hfkwok@um.edu.mo; 2 Institute of Translational Medicine, Faculty of Health Sciences, University of Macau, Taipa, Macau, China

Targeted therapy has been a very hot research topic in the last decade. It focuses on specific medications for treatment of particular diseases, such as cancer, diabetes, heart disease, etc. One of the most exciting recent developments in targeted therapies is the isolation of disease-specific molecules from natural resources, such as animal venoms and plant metabolites/toxins, to use as templates for new drug motif design. 

This Special Issue of Toxins includes three recent advanced research studies related to bee venoms as potential medicinal therapy in different aspects [1,2,3]. Furthermore, recent advances in bioactive molecules finding from frog skins, mushroom and venom/toxin/immunotoxins for targeted cancer therapy and immunotherapy are discussed [4,5,6,7,8]. The discussion on using novel disease-specific venom-based protein/peptide/toxin along with currently available FDA approved drugs as combinatorial treatment, such as a family of novel types of antimicrobial agents, were also encouraged to be discussed in these contexts. For examples, an overview of some selected promising snake and ant venom-based peptides/toxins potentially able to address the forthcoming challenges in this field were included [9,10]. In addition, four detailed review articles openly discuss the venom proteins/peptides in different species of snake venoms and toad toxins [11,12,13]; moreover, plant toxins from Bouganin naturally targeted, as immunotoxins [14], mammalian receptors and demonstrated high specificity and selectivity towards defined ion channels of cell membranes and receptors. 

To sum up, all research and review articles proposing novelties or overviews, respectively, were successfully and carefully selected in this Special Issue after rigorous revision by the expert peer reviewers. As a guest editor, I would like to express my deep appreciation to all the selfless and fair reviewers.

## References

[1] Shin D., Choi W., Bae H. (2018). Bee venom phospholipase A2 alleviate house dust mite-induced atopic dermatitis-like skin lesions by the CD206 mannose receptor. Toxins.

[2] Kim Y., Lee Y.W., Kim H., Chung D.K. (2019). Bee Venom Alleviates Atopic Dermatitis Symptoms through the Upregulation of Decay-Accelerating Factor (DAF/CD55). Toxins.

[3] Frangieh J., Salma Y., Haddad K., Mattei C., Legros C., Fajloun Z., El Obeid D. (2019). First Characterization of The Venom from *Apis mellifera syriaca*, A Honeybee from The Middle East Region. Toxins.

[4] Najar D., Haraldsson B., Thorsell A., Sihlbom C., Nyström J., Ebefors K. (2018). Pharmacokinetic Properties of the Nephrotoxin Orellanine in Rats. Toxins.

[5] Tan Y., Chen X., Ma C., Xi X., Wang L., Zhou M., Burrows J., Kwok H., Chen T. (2018). Biological Activities of Cationicity-Enhanced and Hydrophobicity-Optimized Analogues of an Antimicrobial Peptide, Dermaseptin-PS3, from the Skin Secretion of *Phyllomedusa sauvagii*. Toxins.

[6] Rust A., Shah S., Hautbergue G., Davletov B. (2018). Burkholderia Lethal Factor 1, a Novel Anti-Cancer Toxin, Demonstrates Selective Cytotoxicity in MYCN-Amplified Neuroblastoma Cells. Toxins.

[7] Lin Y., Hu N., He H., Ma C., Zhou M., Wang L., Chen T. (2019). A Hylarana latouchii Skin Secretion-Derived Novel Bombesin-Related Pentadecapeptide (Ranatensin-HLa) Evoke Myotropic Effects on the in vitro Rat Smooth Muscles. Toxins.

[8] Müller F., Cunningham T., Beers R., Bera T., Wayne A., Pastan I. (2018). Domain II of Pseudomonas Exotoxin Is Critical for Efficacy of Bolus Doses in a Xenograft Model of Acute Lymphoblastic Leukemia. Toxins.

[9] Monteiro D., Selistre-de-Araújo H., Tavares D., Fernandes M., Kalinin A., Rantin F. (2017). Alternagin-C (ALT-C), a Disintegrin-Like Cys-Rich Protein Isolated from the Venom of the Snake *Rhinocerophis alternatus*, Stimulates Angiogenesis and Antioxidant Defenses in the Liver of Freshwater Fish, *Hoplias malabaricus*. Toxins.

[10] Guzman J., Téné N., Touchard A., Castillo D., Belkhelfa H., Haddioui-Hbabi L., Treilhou M., Sauvain M. (2018). Anti-Helicobacter pylori Properties of the Ant-Venom Peptide Bicarinalin. Toxins.

[11] Li L., Huang J., Lin Y. (2018). Snake venoms in cancer therapy: Past, present and future. Toxins.

[12] Qi J., Zulfiker A., Li C., Good D., Wei M. (2018). The development of toad toxins as potential therapeutic agents. Toxins.

[13] Abidin Z., Asnawi S., Lee Y.Q., Othman I., Naidu R. (2019). Malaysian Cobra Venom: A Potential Source of Anti-Cancer Therapeutic Agents. Toxins.

[14] Bortolotti M., Bolognesi A., Polito L. (2018). Bouganin, an Attractive Weapon for Immunotoxins. Toxins.

